# Extracellular Vesicles as Carriers of Non-coding RNAs in Liver Diseases

**DOI:** 10.3389/fphar.2018.00415

**Published:** 2018-04-24

**Authors:** Junfa Yang, Changyao Li, Lei Zhang, Xiao Wang

**Affiliations:** ^1^School of Pharmacy, Anhui Medical University, Hefei, China; ^2^Anhui Province Key Laboratory of Major Autoimmune Diseases, Anhui Institute of Innovative Drugs, Hefei, China; ^3^The Key Laboratory of Anti-inflammatory and Immune Medicines, Ministry of Education, Anhui Medical University, Hefei, China; ^4^Department of Radiology, The First Affiliated Hospital of Anhui Medical University, Hefei, China

**Keywords:** non-coding RNAs, mRNAs, EVs, liver disease, DNA

## Abstract

Extracellular vesicles (EVs) are small membranous vesicles secreted from normal, diseased, and transformed cells *in vitro* and *in vivo*. EVs have been found to play a critical role in cell-to-cell communication by transferring non-coding RNAs (ncRNAs) including microRNAs (miRNAs), long ncRNAs (lncRNAs) and so on. Emerging evidence shows that transferring biological information through EVs to neighboring cells in intercellular communication not only keep physiological functions, but also participate in the pathogenesis of liver diseases. Liver diseases often promote release of EVs and/or in different cargo sorting into these EVs. Either of these modifications can promote disease pathogenesis. Given this fact, EV-associated ncRNAs, such as miR-192, miR-122 and lncRNA-ROR and so on, can serve as new diagnostic biomarkers and new therapeutic targets for liver disease, because altered EV-associated ncRNAs may reflect the underlying liver disease condition. In this review, we focus on understanding the emerging role of EV-associated ncRNAs in viral hepatitis, liver fibrosis, alcoholic hepatitis (AH), non-alcoholic steatohepatitis (NASH) and hepatocellular carcinoma (HCC) and discuss their utility in biomarker discovery and therapeutics. A better understanding of this multifaceted pattern of communication between different type cells in liver may contribute to developing novel approaches for personalized diagnostics and therapeutics.

## Introduction

Understanding the intercellular communication is rapidly becoming the new frontier in multi-cellular organisms. It is now widely accepted that cell-to-cell communicate not only by direct contact or soluble factors but also through released cell membrane fragments known as extracellular vesicles (EVs) (Howlett and Bissell, 1993; Lee et al., 2011). EVs are membrane-bound vesicles that are released by normal and transformed cells into their environment. Although EVs were first identified in the late 1970s, it has only recently served as the points of focus in the scientific community (Penfornis et al., 2016). Increasing evidence have showed that EVs can be released by most cell types and detected all major bodily fluids, including blood, urine, bile, saliva, breast milk, as well as in cirrhosis associated ascites (Bobrie et al., 2011; Colombo et al., 2014; Yanez-Mo et al., 2015). Emerging studies have also identified that EVs can serve as a unique vehicle for the release of soluble and insoluble molecules and was associated with several physiological and pathophysiological processes, such as inflammation, immune modulation, neurological diseases, cancer and liver diseases (Cossetti et al., 2014; Hazan-Halevy et al., 2015; Polanco et al., 2016; Wong et al., 2016). EVs can interact with the recipient cells through signaling pathways. Actually, during the process of their biogenesis, EVs acquire repertoire of bioactive cargo including membrane-bound proteins, bioactive metabolites, lipids, DNA, microRNA (miRNA), mRNA, along with other regulatory RNAs (**Table 1**) (Hoshino et al., 2015; Zomer et al., 2015; Keerthikumar et al., 2016). However, the mechanism of EVs loading is unclear. Additionally, endocytosis is the main way to EV uptake in more cell types, including clathrin-dependent endocytosis and clathrin-independent pathways. Lipid and protein interactions contribute to EV uptake (Mulcahy et al., 2014). Following this discovery, we review the role of non-coding RNAs (ncRNAs) carried by EVs in diagnosis and the assessment of liver disease, and how to use them as new diagnostic biomarkers and therapeutic targets.

**Table 1:** The composition of EVs.

Components	EVs cargo	Citation
Lipids	Cholesterol, sphingomyelin, phosphatidylserine, ganglia glycosides	Povero et al., 2014; Becker et al., 2016
Nucleic acids	DNA (e.g., mtDNA), mRNA, non-coding RNAs (e.g., miRNA, lncRNA), viral RNA (e.g., HBV-RNA), tRNA	Cossetti et al., 2014; Wendler et al., 2017
Proteins	RabGTPases, CD9, flotillin, annexin, TSG101, CD63, CD81	Yanez-Mo et al., 2015; Yang et al., 2016

### Non-coding RNAs (ncRNAs)

As is well-known, ncRNAs are usually divided into two main classes according to their size: (1) short ncRNAs (<200 nt), such as miRNAs, endogenous small interfering RNA (siRNA) and piwi-interacting RNA (piRNA); (2) long ncRNAs (lncRNA;>200 nt) (Mercer et al., 2009; Prensner and Chinnaiyan, 2011; Raposo and Stoorvogel, 2013). Although ncRNAs are unable to encode protein, they have specific biological function of regulating the levels of proteins and mRNAs of target genes, interacting with proteins to disturb their function, and interacting one with each other to finely tune their expression (Ling et al., 2013) (**Table 2**).

**Table 2:** EV-associated ncRNAs in liver disease.

ncRNAs	Expression	Liver disease	Function	Citation
miR-122	Up	HCV	Biomarker	Bihrer et al., 2011
miR-192	Up	AH	Diagnostic markers	Ghidini and Braconi, 2015
miR-21	Up	HCC	Inhibitor apoptosis	Gomaa et al., 2008
miR-18a	Up	HCC	Promotes cell proliferation	Sohn et al., 2015
miR-221	Up	HCC	Promotes growth and invasion of cells	Sohn et al., 2015
miR-222	Up	HCC	Promoters proliferation	Sohn et al., 2015
linc-VLDLR	Up	HCC	Promotes chemoresistance	Giroud and Scheideler, 2017
linc-RoR	Up	HCC	Promotes tumor	Loewer et al., 2010; Nath and Szabo, 2012
miR-718	Down	HCC	Inhibitor proliferation	Sugimachi et al., 2015
miR-199a	Down	HCV	Inhibitor of HCV transmission	El-Abd et al., 2015
miR-30a	Up	AH	Potential diagnostic markers	Momen-Heravi et al., 2015
miR-122	Up	NASH	Biomarker	Miyaaki et al., 2014
miR-182	Up	AH	Increases inflammatory mediators	Blaya et al., 2016
miR-193b	Down in HCC, up in HCV-HCC	HCC HBV-HCC	Diagnostic tool/biomarker	Braconi et al., 2010; Mao et al., 2014

The miRNAs, such as miR-145 and miR-122, are the most broadly reported ncRNA in the literature, since the first small ncRNA lin-4, in *C*. *elegans*, was described in 1993 (Lee et al., 1993; Wightman et al., 1993). miRNAs are a broad class of endogenous short ncRNAs that are approximately ∼22 nucleotides in length and can regulate gene expression by silencing or post-transcriptionally (Ambros, 2004; Bartel, 2004; Malizia and Wang, 2011; Yates et al., 2013). Of note, miRNAs were reported to target many genes and involved in regulation cell proliferation and differentiation, migration, apoptosis, and modulation of the host response to viral infection (Bartel, 2004; Salmanidis et al., 2014). Alterations in miRNAs expression profiles have been shown to be associated viral hepatitis, hepatobiliary malignancies, non-alcoholic fatty liver disease, acute liver injury from acetaminophen and liver fibrosis (Jiang et al., 2010; Cermelli et al., 2011; Starckx et al., 2013; Szabo and Bala, 2013; Yao et al., 2017). Interestingly, miRNAs not only exert their function intracellularly, but also can be exported from cells in the extracellular space via EVs or bound to proteins (Vickers et al., 2011; Boon and Vickers, 2013). Furthermore, increasing evidence suggests that EVs can protect miRNAs from the degradation of RNAses, thus elevate the stability of miRNAs (Boon and Vickers, 2013). It is therefore not surprising that miRNAs have been considered as a novel class of biomarkers in liver diseases (Gui et al., 2011; Waidmann et al., 2012; do Amaral et al., 2017). Recently, Russo et al. (2017) demonstrated that patients with drug-induced liver injury (DILI) showed enhancive serum levels of miR-122, -1246, -4270, -4433, -4463, -4484, -4532, and pre-miR-4767 as well as reductive serum levels of miR-455-3p, -1281, and pre-miR-4274 compared to controls. Meanwhile, two liver-enriched miRNAs in EVs have been suggested to be more informative DILI biomarkers than ALT alone (Russo et al., 2017). Another study suggests that miR-181b in serum from 22 patients with liver cirrhosis was elevated. In turn, miR-181b promoted HSC-T6 proliferation by targeting the cell cycle regulator p27 (Wang et al., 2012). A panel of seven plasma miRNAs (miR-122, miR-192, miR-21, miR-223, miR-26a, miR-27a, and miR-801) was shown to have a high-diagnostic accuracy of HCC (Ghidini and Braconi, 2015). Furthermore, recent studies have revealed that the levels of miRNA-122 were remarkably increased and almost exclusively detected in circulating EVs but not in the non-EV fractions in HCC (El-Abd et al., 2015).

Long ncRNAs are the most heterogeneous class of non-protein-coding RNAs with length from 200 nt to 100 kb. Although, the lncRNAs are expressed at low levels compared with mRNAs, they have been shown to exhibit tissue or cell type-specific expression (Ghidini and Braconi, 2015). Similarly to mRNAs, most lncRNAs have many features in common with mRNAs, as transcription by RNA polymerase II, polyadenylation and splicing mechanisms (Guttman et al., 2009; Binder et al., 2011). With the rapid growth of lncRNA study, the landscape of lncRNAs has been unveiled by the rapid development of deep sequencing technology along with the development of bioinformatics tools (He et al., 2014a). According to their genomic proximity to protein-coding genes, lncRNAs have been divided into four categories: sense lncRNAs, antisense lncRNAs, bidirectional or divergent lncRNAs and intergenic lncRNAs (Tang et al., 2013; Rodriguez-Malave and Rao, 2016). Due to lncRNA complex structure features, it is also offers multiple possibilities for interactions with DNA, RNA, and/or protein (Van Roosbroeck et al., 2013). Noteworthy, the function of lncRNAs was associated with their cellular localization (Zhang et al., 2014). In the nucleus, some lncRNAs can influence chromatin architecture through interacting with chromatin-modulating proteins which can facilitate their recruitment and/or combine to chromatin, thereby regulating transcriptional activity (Gonzalez et al., 2015; Scarola et al., 2015; Liu et al., 2017; Wang and Dostie, 2017). In the cytoplasm, lncRNAs seem to bind to the ribosome. Emerging studies have also identified that lncRNAs can act as molecular decoys for proteins or miRNAs (Carlevaro et al., 2016). Likewise, lncRNAs, act like miRNA sponges, modulate the microRNome by binding one or multiple miRNAs (Giroud and Scheideler, 2017). To our knowledge, lncRNAs has been shown to play major roles and biomarkers in disease genesis and diagnosis. Growing evidence is now suggesting that the lncRNAs was associated with pathophysiological conditions, including cancer, diabetes, liver disease and some other complex disorders (Reddy et al., 2014; Gooding et al., 2017; Shan et al., 2017). For example, ISR2, ISR8, lncISG15, and BISPR are upregulated in HCV-infected livers and cultured cells through the IFN signaling pathway (Barriocanal et al., 2014; Carnero et al., 2014). Dysregulated lncRNA-MALAT1 expression is associated with inflammation and fibrosis in NASH (Leti et al., 2017). MEG3 levels were decreased in CCl_4_-induced mouse liver fibrosis models, human fibrotic livers and human hepatic stellate cell (HSC) lines LX-2 cells after treatment with TGF-β1 (He et al., 2014b). These studies showed that lncRNAs have been acted as a novel class of biomarkers for many liver diseases. Noteworthy, one major finding is that most lncRNAs in EVs show a specific pattern of expression across several stages of development of liver diseases (Ghosal et al., 2013). lncRNA in serum and exosomes as a potential biomarker in the HCV-related HCC (Zhang et al., 2018). Recent studies showed that several lncRNAs significantly increased in HCC. These lncRNAs might contribute to oncogenesis during the development of HCC. For example, linc-RoR can regulate cell viability of HCC cells during hypoxia (Takahashi et al., 2014a).

To conclude, ncRNAs can be released into the extracellular medium associated with lipoproteins or encapsulated in EVs, thus participating in cell-to-cell communication (Bobrie et al., 2011). These findings add attention to explore the functions of ncRNAs, especially EV-associated ncRNAs, because they imply that the message delivered by each EV-associated ncRNAs may be amplified according to the expression of their isoforms.

### EV-Associated Non-coding RNAs

**FIGURE 1:**
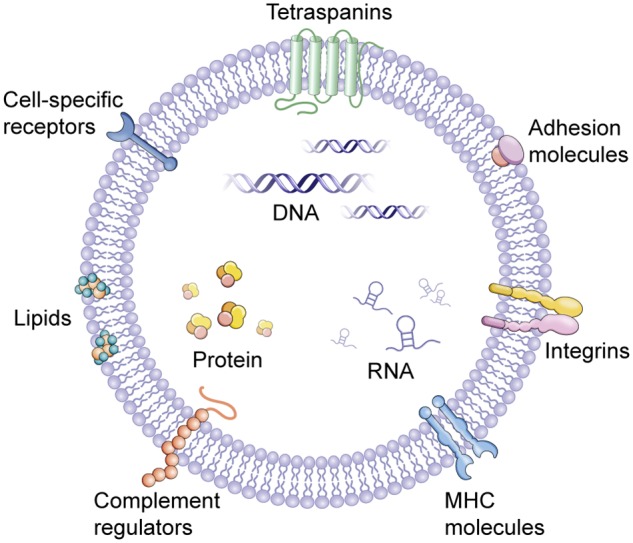
Extracellular vesicles (EVs). EVs contain several types of molecules, proteins, DNA, mRNA, lncRNA, and miRNA, some of which are selectively enriched and specific to cell of origin. MHC, major histocompatibility complex.

**FIGURE 2:**
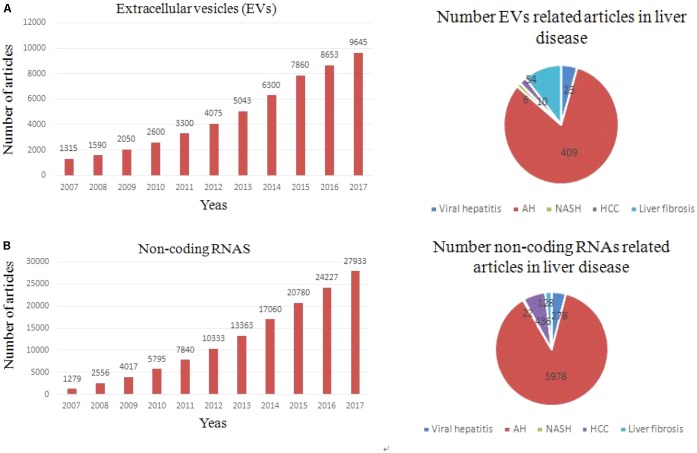
Trends of non-coding RNAs and EVs related articles, since 2000. **(A)** Exponential growth in the number of EVs-related articles (upper left panel) and number of articles related to each type of liver fibrosis (upper right panel). **(B)** Exponential growth in the number of non coding RNAs-related articles and total number of articles related to each liver fibrosis.

The word “EV” is actually a collective term that refers to a series of lipid bilayer membrane-bound organelles that are released by cells into their environment (**Figure 1**). In the last years, the discovery of EVs has represented a revolution and a paradigm shift (**Figure 2A**). EVs are heterogeneous in size and are released from nearly all cells under both physiological and pathological conditions (Lee et al., 2011). Currently, more specific nomenclature for EVs bases on their size, biogenesis, and secretion mechanisms including exosomes, ectosomes, microvesicles, apoptotic bodies and oncosomes (Yanez-Mo et al., 2015). Nevertheless, exosomes and microvesicles are the most commonly used as the term ‘EVs’. Exosomes(40–100 nm diameter) are originated from the inward and reverse budding of an endosomal membrane and are released into the extracellular space following fusion of multivesicular bodies (MVBs) with the plasma membrane (Colombo et al., 2014; Robbins and Morelli, 2014). Microvesicles (100–1,000 nm diameter), larger than exosomes, are cell surface-derived EVs that directly bud from the plasma membrane (Camussi et al., 2011).

In 2007, EVs was first supposed to be an important mediator of many biological events through transferring genetic information to target cell (Valadi et al., 2007). Nowadays, growing evidence showed that EVs play a key role in intercellular communication through delivery their cargo (proteins, RNAs, and lipids) (Ekstrom et al., 2012). The recognition that EVs released from a cell can be absorbed by another and transfer their cargo (Penfornis et al., 2016). The EV cargo partly reflects the cells of origin, and especially exosomes have been shown to carry different RNA species compared to their parental cells (Skog et al., 2008; Ekstrom et al., 2012; Nolte-’t Hoen et al., 2012). In fact, transfer of EV cargo is one of mechanisms about intercellular communication (Mathivanan et al., 2010; Ling et al., 2013). It is known that unprotected ncRNAs are easily degraded by the RNases in blood. However, EVs can protect ncRNAs from degradation and maintains their integrity and activity in the circulation. According to an exosome database, ExoCarta, 4,563 proteins, 194 lipids, 1,639 mRNAs, and 764 miRNAs have been identified in exosomes which were released from different cells (Kosaka et al., 2013; Yang et al., 2016). Recent findings suggest that plasma (HULC) lncRNA expression in HCC is significantly higher than in the healthy controls (Xie et al., 2013). Growing researches suggested that many types of cells in the liver have the capacity to secrete EVs under *in vitro* conditions, including hepatocytes, HSCs, Kupffer cells, cholangiocytes, and other immune cell (Rodriguez-Suarez et al., 2014; Chen et al., 2015; Szabo and Momen-Heravi, 2017). Multiple effects of EVs-associated ncRNAs in cell-to-cell communication in the liver will be illustrated as bellow. Changes in the transcriptomic and proteomic EV content (ncRNAs) have been reported in different liver disease, pointing out their potential value as non-invasive biomarker (**Figure 2B**). Manifestation of a role for EVs-associated ncRNAs in liver disease pathogenesis will support their potential use as disease biomarkers. The ability to selectively manipulate ncRNAs in EVs through either exogenous or endogenous approaches offers further opportunities for their use in disease therapeutics.

## Ev-associated Non-Coding RNAs in Liver Diseases

### Viral Hepatitis

Viral hepatitis is caused by a variety of hepatitis viruses, which can arise as a consequence of liver disease. Many studies showed that the EVs derived from a variety of cells play multiple roles in the development of viral hepatitis, including virus transmission, host immune regulation and micro-environment manipulation (Ramakrishnaiah et al., 2013). Hepatitis C virus (HCV)-infected hepatocytes-derived EVs have the function of transmitting HCV infection (Bukong et al., 2014;Shen et al., 2017). Interestingly, many studies provided insights into the important role of miRNAs in EVs in the development of viral hepatitis. HCV RNA is associated with miR-199a and miR-221 in HCV infection hepatocytes. MiR-199a in HCV patients was significantly lower than in healthy controls (Shigehara et al., 2011; Shrivastava et al., 2015). EVs loaded with miR-199a or miR-221 significantly suppressed EV-associated HCV transmission to naive cells. A contributing function of EVs in viral hepatitis is further highlighted by studies showing that functional ncRNAs in EVs largely contributed to the suppression of HCV RNA replication, such as miR-199a and miR-145, resulting in the down-regulation of intracellular HCV RNA and the release of HCV (Waidmann et al., 2012; Qian et al., 2016). Additionally, EVs enhance the response of innate immune to HCV-infected hepatocytes. Of note, increasing evidence suggests that EVs derived from human liver endothelial cells inhibit viral replication through autocrine interferon signaling (Giugliano et al., 2015; Shrivastava et al., 2015). Hence, identification specific role of ncRNAs in EVs to viral infection and transmission will advance our understanding of viral hepatitis mechanisms, and potentially yield targets for developing innovative preventive or therapeutic regimens.

### Liver Fibrosis

Liver fibrosis is characterized by extensive deposition of extracellular matrix (ECM) and an activation of HSCs resulting from a protracted wound-healing response (Schuppan and Kim, 2013; Fagone et al., 2016). HSCs, previously known as vitamin A-storing cells or Ito cells, are primary source of ECM components in the development of liver fibrosis (Kisseleva and Brenner, 2006, 2007). Following liver fibrosis of any cause, HSCs were activated, which is the transition of quiescent HSCs into proliferative, fibrogenic, and contractile myofibroblasts (Schuppan and Kim, 2013). Of note, it has been recognized that suppression of the activation of HSCs can prevent and treat liver fibrosis (Lee and Friedman, 2011). Some studies have reported that pathological HSCs migration can be regulated by endothelial cell-derived EVs during liver fibrosis (Wang et al., 2015). The study found that EVs induced HSCs migration through EVs adhesion. Additionally, many studies found that EV-associated ncRNAs could also regulate the activation and proliferation of HSCs through regulating anti- and pro-fibrotic gene expression (Royo and Falcon-Perez, 2012; Matsuura et al., 2016). In a recent study, connective tissue growth factor (CTGF) promotes fibrogenesis in HSCs. miR-214 can specifically targets the 3′-UTR regions of CTGF in activated HSCs. Interestingly, miR-214 in EVs derived from HSCs suppressed CTGF3′-UTR region activity and thus inhibit the expression of CTGF. Nonetheless, the same group demonstrated afterward that CTGF is also loaded into EVs and transferred between HSCs, resulting in an increase in the level of alpha smooth muscle in the recipient HSCs (Charrier et al., 2014; Chen et al., 2014; Lee et al., 2017). Additionally, studies found that EVs derived from hepatocyte regulate HSCs via miR-128-3p targeting PPAR-γ. miRNAs in Hepatocyte-derived EVs are known inhibitors of PPAR-γ expression with miR-128-3p being the most effectively transferred. Furthermore loss- and gain-of-function studies identified miR-128-3p as a central modulator of the effects of EVs on PPAR-γ inhibition and HSC activation (Povero et al., 2015). Furthermore, pathway analysis suggested that low levels of let-7 in EVs may influence hepatic fibrogenesis through activation of transforming growth factor β signaling in HSCs (Matsuura et al., 2016). Hence, ncRNAs derived from EVs serve as fibrosis regulators and may be considered as biomarkers of liver fibrosis.

In addition, studies found that EVs derived from platelets or granulocytes in patients with sepsis have function of promote coagulation and suggested thrombin generation, in proportion to the severity of the disseminated intravascular coagulopathy (Nieuwland et al., 2000; Owens and Mackman, 2011). However, many kinds of studies have demonstrated that coagulation activation enhances liver fibrosis (Valla, 2008; Dhar et al., 2017). Therefore, EVs might also promote fibrogenesis. Thence, ncRNAs in EVs may act as a key regulator in liver fibrosis s by promoting migration and activation of HSCs, in addition to their known anti-fibrosis function.

### Alcoholic Hepatitis

Alcoholic hepatitis (AH) is an acute hepatic inflammation accompanied with intrinsical morbidity and mortality and increasing health problems across the world. AH is a result of the complex interactions between ethanol metabolism, inflammation, and innate immunity on the liver (Orman et al., 2013). Currently the most commonly used indicators in liver damage are the enzymatic activity of alanine aminotransferase (ALT) and aspartate aminotransferase (AST) in blood. However, serum elevated ALT and AST are poor specificity for liver disease (Kim et al., 2008). Several recent studies have focused on the effect of ncRNAs in EVs on AH. Recent studies showed that EVs could serve as potential diagnostic markers for AH (Momen-Heravi et al., 2015). Macrophage phenotype can be regulated by EVs derived from hepatocyte and monocyte, thereby EVs enhance inflammation in AH. Interestingly, previous studies demonstrated that several ncRNAs have been thought to be linked to EVs-associated cell-to-cell signaling, including miR-192 and miR-30a (Momen-Heravi et al., 2015).

Several reports have confirmed that the miRNAs cargo within EVs derived from sera of alcohol-fed mice are potential novel biomarkers in AH. MiR-192 and miR-30a were considered as EV cargos that were significantly increased in the circulation of subjects with AH (Momen-Heravi et al., 2015). Additionally, a growing body of evidence shows that the protein cargoes within EVs derived from alcohol-treated hepatocytes stimulate macrophage activation via Hsp90. For example, CD40 ligand was considered as an EV cargo that could induce the activation and infiltration of macrophages in AH (Verma et al., 2016). These findings indicate that cells in disease conditions promote the secretion of EVs containing unique molecules, and lead to diverse pathophysiological events. Importantly, studies on EV-associated ncRNAs have also been carried out human specimens that focus on the potential biomarkers of EV-associated ncRNAs in AH. Studies suggested that EVs is significantly increased in alcohol-fed mice and human subjects with AH. miRNAs of exosomes were significantly up-regulated in alcohol-fed mice compared to pair-fed mice and had valuable diagnostic values including miR-192 and miR-30a. Consistently, miRNA-30a and miRNA-192 were increased significantly in exosomes derived from plasma of AH patients (Momen-Heravi et al., 2015). Indeed, further studies are required to understand if EVs-associated ncRNAs has a biomarker value in the diagnosis and prognosisof AH.

### Non-alcoholic Steatohepatitis

Non-alcoholic fatty liver disease (NAFLD) is a dominant cause of chronic liver disease around the world. NAFLD has become a severe health issue and it can progress toward a more severe form of the disease, the non-alcoholic steatohepatitis (NASH) (Alazawi et al., 2010). NASH is characterized by lobular and portal inflammatory infiltrates of monocyte-derived macrophages, neutrophils and lymphocytes and so on. Over the last few years, cell-derived EVs have been considered as effective regulators in cell-to-cell communication that transfer several bioactive molecules in target cells, regulating the pathogenesis and progression of NASH (Povero et al., 2013). Study results demonstrated that the levels of blood EVs were increased during the progression of NASH and were strongly associated with some key outcomes of NASH in experimental models of NASH (Povero et al., 2014). The bioavailability of EVs in biofluids is only one of the fascinating aspects of these effective “messengers.” Indeed, the composition of EVs implies a special potentiality for biomarker development. As a proof of this, ncRNAs packaged in EVs have increasingly manifested great potential to meet several criteria for being considered as good biomarkers (Lai et al., 2014; Becker et al., 2015). They found that ncRNAs in EVs were significantly different in patients with NASH compared to control, such as miR-192 and miR-122. Insights into the potentiality of ncRNAs in EVs as biomarkers were provided by the observation that the expression of miRNA-122 and miRNA-192 were increased in circulating exosomes from advanced stage NAFLD patients compared to those from early stage NAFLD patients (Lee et al., 2017). The increase of miRNAs from stressed or damaged hepatocytes during NAFLD progression may be related to the decreased expression level of miR-122 found in the livers of patients with advanced NAFLD and early stage hepatocarcinogenesis from NASH, as recently reported (Esau et al., 2006; Cheung et al., 2008; Takaki et al., 2014). Therefore, this miRNA level in EVs might be useful as a biomarker for the diagnosis of advanced NAFLD or NASH. Consequently, these findings provide important support to the development of biomarkers. EV-associated ncRNAs can be used for biomarkers to make novel and non-invasive diagnostic strategies for NASH.

### Hepatocellular Carcinoma (HCC)

Hepatocellular carcinoma (HCC) is the most common malignancy and the third leading cause of cancer-related death worldwide. HCC has been certificated to be highly refractory to treatment (Gomaa et al., 2008; Alazawi et al., 2010; Forner et al., 2018). The crosstalk of the tumor micro-environment with cancer cells is essential for tumor progression (Tlsty and Coussens, 2006). Previous studies have demonstrated that EVs play a regulatory role in cell-to-cell communication, including cancer cells. Additionally, it was also shown that tumors prepare their own tumor niches through the release of EVs (Becker et al., 2016; Wendler et al., 2017). EVs when were absorbed by HCC cells promote their migration, invasion, and proliferation. Furthermore, many studies have evaluated the role of EVs mediated ncRNAs in hepatocarcinogenesis and tumor progression (Wang et al., 2018). For instance, bona fide miRNA biomarker in serum exosomes predicts HCC recurrence after liver transplantation (LT). The studies found that miR-718 showed significantly different expression in the serum exosomes of HCC cases with recurrence after LT compared with those without recurrence (Sugimachi et al., 2015). Of note, ncRNAs were shown to have diagnostic, prognostic, and therapeutic potential in HCC. EVs mediated ncRNAs transfer between HCC cells was considered as an important regulator to local spread and multifocal growth, such as miR-584, miR-517c and miR-378 (Kogure et al., 2011). The activation of MAPK pathway could induce the epithelial mesenchymal transition and up-regulate cyclin-dependent kinase 2 (CDK2) and MMP2 expression to promote cell proliferation and metastasis. Interestingly, EVs mediated miR-320a could function as anti-tumor miRNAs by binding to its direct downstream target PBX3 to suppress HCC cell proliferation, migration and metastasis. The miR-320a-PBX3 pathway inhibited tumor progression by suppressing the activation of the MAPK pathway in HCC (Zhang et al., 2017). Hence, these observations indicate that EV-associated ncRNAs act as HCC modulators and may serve as a biomarker of HCC.

On the other hand, growing studies showed that EV-mediated lncRNAs can regulate gene expression via various mechanisms. EV–mediated transfer of lncRNA as a mechanism by which tumor cells can modulate their local cellular environment (Kogure et al., 2013; Conigliaro et al., 2015). These results demonstrated that lncRNAs can also act as biomarkers in HCC. Furthermore, HCC-derived EVs may enhance the host antitumor immune responses (Lv et al., 2012). EV-associated shuttle of lncRNAs specifically lnc-VLDLR and lnc-ROR enhance chemoresistance of malignant cells. The antitumor ability of natural killer (NK) cells can be increased heat shock proteins (HSPs)-harboring exosomes derived from HCC, thus promoting HCC immunotherapy. The study provides evidence that the expression of lnc-VLDLR was up-regulated by multiple anticancer agents in HCC cells and EVs derived from HCC cells (Takahashi et al., 2014b). Interestingly, chemotherapy-mediated cell death can be decreased by these EVs, along with up-regulation of lnc-VLDLR expression in the recipient cells. The knockdown of lnc-VLDLR inhibits cell viability. Therefore, linc-VLDLR was EV-enriched a lncRNA that regulate HCC cell chemoresistance. Collectively, EV-associated ncRNAs play multiple roles in mediating progression, metastasis and thus can be used as a potential therapy for the treatment of HCC. In addition, EV-associated ncRNAs species could be exploited for the non-invasive diagnosis of HCC.

## Summary and Future Prospects

**FIGURE 3:**
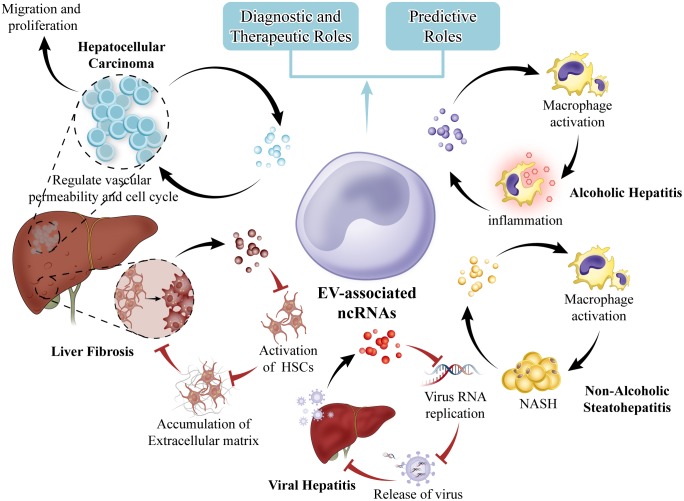
EVs in liver diseases. EVs from hepatocytes can mediate early immune responses in drug-induced liver injury, promote alcoholic and non-alcoholic steatohepatitis through activation of macrophages. EVs from hepatic endothelial cells can inhibit viral progression or virus RNA replication. HCC cell EVs can create a premetastatic niche, educate the tumor stroma, and promote metastasis. EVs can be exploited for therapeutic intervention. Selective EV contents that are specific to cell of origin such as miRNA are potential biomarkers for disease.

In conclusion, we have highlighted several of the most recent and original studies reporting the role of ncRNAs in EVs as link between liver fibrosis, viral hepatitis, NASH, AH and HCC. The association of specific EV constituents with disease offers potential for the use of EVs as biomarkers to diagnose and predict behavior or treatment response in many different types of liver diseases (**Figure 3**). Our knowledge about EVs has been rapidly growing in recent years, along with the analyses of protein, mRNA, ncRNAs and lipid in EVs. We search recently published articles about EV-associated ncRNAs by using PubMed tool. We found that EV-associated ncRNAs are increasingly recognized as essential regulators of biological processes in normal and pathological conditions thus having a high potential to targeted delivery and therapeutic intervention in liver and other diseases. There are evidences that ncRNAs in EVs may sever as or transfer damage-related molecular patterns to recipient cells of the liver micro-environment and coordinate many of the main pathological events occurring concurrently during the liver disease progression. Therefore, the ncRNAs in EVs may become specific biomarkers for liver disease if research will reveal that ncRNAs are indeed differ in different pathologies, and technologies will be advanced to allow the routine detection of the ncRNAs in individual samples. Nevertheless, the information that can be acquired by the detailed detection of EVs-associated ncRNAs is already contributing to understand the pathogenesis of liver diseases, and will do so even more in the future. Our previous researches showed that ncRNAs play regulatory roles in liver fibrosis and tumor. We found that miR-145 inhibits HSCs activation and proliferation by targeting ZEB2 through Wnt/β-catenin pathway. EVs are being developed also as highly multifunctional drug delivery tools with new features. Thus EVs-associated ncRNAs are one of the more promising fields of investigation in liver diseases, with the potential to shed light on pathogenic mechanisms, to reveal long needed biomarkers. It is hoped that these efforts will be soon translated into clinical practice. To achieve this goal, a combination of clinically driven, genetically defined, morphologically classified, and molecular-based study will have to be performed. Multidisciplinary research group with differential background should cooperate to successfully address these issues.

## Author Contributions

LZ and XW designed review idea and took part in the critical revision of manuscript. JY participated in drafting of manuscript; CL participated in the cited articles search.

## Conflict of Interest Statement

The authors declare that the research was conducted in the absence of any commercial or financial relationships that could be construed as a potential conflict of interest.
